# Recent advances in myelodysplasia: update from 2011 ASH annual meeting

**DOI:** 10.1186/1756-8722-5-S1-A4

**Published:** 2012-04-25

**Authors:** Delong Liu

**Affiliations:** 1 Division of Hematology /Oncology, New York Medical College & Westchester Medical Center, Valhalla, NY 10595, USA

## 

Significant progresses have been made in genetic research in MDS. Through RNA interference technology, knock-down of RPS14 recapitulated the pathological process of decreased erythropoiesis [1]. Transgenic expression of RPS14 in 5q- MDS cells rescued the phenotype of insufficient erythropoiesis. This strongly suggests that haploinsufficiency of RPS14 is one of the molecular mechanisms in the pathogenesis of 5q- MDS. SF3B1 is a core component of RNA splicesome and involved in the regulation of the mitochondrial pathway. In a study of 533 patients (pts) with MDS, 150 (28.1%) was found to have SF3B1 gene mutation, which has a positive predictive value of 97.7% for RARS and correlates well with better overall survival (OS) and lower risk for AML transformation [2,3].

Hypomethylating therapy represents a significant milestone in myelodysplasia (MDS) management. TET2, IDH1/2, and DNMT3A are regulators of DNA methylation [4]. EZH2 and UTX were found to be involved in the histone H3K26 and H3K27 methylation. ASX1 was found to be deleted in 11-15% of MDS pts. In a study of 88 pts with MDS, mutations of DNMT3A, IDH1/2, and TET2 were found to be correlated with responses to azacytidine /decitabine(64% in mutated vs 35% wild type, P=0.01) [5]. microRNA-21 may also serve as a biomarker for therapy response to hypomethylating agents in MDS. In a study of 63 pts, lower level of serum miR-21 correlated with higher PFS and OS (p 0.003 and 0.001, respectively) [6].

In terms of therapy, azacytidine (75 mg /m2 x 5) was studied in combination with lenalidomide (10 mg x 21) in 36 refractory pts (IPSS=>1.5) . Overall response rate (ORR) was 71% (CR 40, PR 31) [7]. Azacytidine (75 mg /m2 x 5 ) was also studied in combination with vorinostat (200 mg TID x 5) in 30 untreated MDS pts with poor clinical status (Cr>=1.5, Bilirubin >=2.0). ORR was 30% (CR 27%) and OS was 7 months in these high-risk pts (expected pre-therapy OS <60 days) [8].

Several novel agents were also reported. Oral decitabine was reported in a phase I bioavailability trial [9]. The oral decitabine has a bioavailability of 3.9 to 14%. Oral doses of 30- 240 mg in MDS patients had similar safety profiles to that of the 20 mg /m2 IV administration. RAP-536 was found to promote erythropoiesis in a mouse model through an EPO-independent pathway [10]. IRAK1 is a serine /threonine kinase. miRNA-146a –deficient mice had IRAK1 overexpression and developed MDS-like phenotype. Phosphorylated IRAK1 was higher in MDS patients. A small molecule inhibitor of IRAK1 was studied in cell lines and in MDS sample cells. Increased apoptosis was seen in these cells. Interestingly, IRAK1 inhibitor spared the normal CD34+ cells [11].

Iron chelation therapy is increasingly used in MDS pts, especially when MDS patients are living longer with the current therapies. Retrospective analysis of iron chelation therapy in MDS pts was reported from Italy and Canada [12,13]. However, the efficacy in MDS cannot be clearly ascertained since there is no randomized prospective study specifically addressing this issue.
